# Nuclear Architecture in the Nervous System: Development, Function, and Neurodevelopmental Diseases

**DOI:** 10.3389/fgene.2018.00308

**Published:** 2018-08-06

**Authors:** Kenji Ito, Takumi Takizawa

**Affiliations:** ^1^Department of Pediatrics, Graduate School of Medicine, Gunma University, Maebashi, Japan; ^2^Division of Stem Cell Pathology, Center for Experimental Medicine and Systems Biology, Institute of Medical Science, University of Tokyo, Tokyo, Japan

**Keywords:** chromatin, neuron, glia, differentiation, development, epigenetics, nuclear architecture, gene positioning

## Abstract

Decades of study have shown that epigenetic regulation plays an important role in neural development and function. Several layers of epigenetic mechanisms control functions of the eukaryotic cell nucleus, a well-organized subcellular organelle with distinct compartments: chromatin, its related architectural proteins, and nuclear bodies. As these components function together in the epigenetic regulation of cellular development and functions, they are collectively termed nuclear architecture. In the nervous system, dynamic rearrangement of nuclear architecture correlates with alteration of transcription programs. During maturation and upon depolarization, neurons undergo a reorganization of nuclear architecture that alters gene expression programs. As such changes allow for specialized functions, including learning and memory, nuclear architecture is distinct among cell types. Studying nuclear architecture of neurons may uncover cell-division-independent mechanisms of global and local changes to nuclear architecture. We herein review recent research concerning nuclear architecture in the nervous system and will discuss its importance to the development, maturation, function, and diseases of the nervous system.

## Introduction

Eukaryotic nuclei stably retain large genomic DNA in a compact space without compromising its ability to read necessary information from genomic DNA to create diverse cell types in the right place at the right time. Uncovering how a cell selects and expresses appropriate genes to acquire its identity is central to understanding the mechanisms underlying the physiological functions of a cell.

Epigenetic alterations play pivotal roles in gene expression and cellar functions in the nervous system (Hirabayashi and Gotoh, 2010). Major epigenetic alterations, such as DNA methylation and histone posttranscriptional modifications, correlate with the structure of chromosomes and chromatin. The former is arranged within the interphase cell nucleus in a cell type and developmental stage-dependent manner (Misteli, 2007). Subdomains of chromosomes and genes in the nucleus also non-randomly change their positions during brain development (Takizawa and Meshorer, 2008). Akin to the genome, nuclear bodies, which occupy functionally/morphologically separate sub-nuclear regions such as the nucleoli, Cajal bodies (CBs), and promyelocytic leukemia(PML) bodies, are also non-randomly arranged (Takizawa and Meshorer, 2008). The organization of these structures is associated with gene expression. The non-random assembly of genomes and nuclear bodies are collectively referred to as “nuclear architecture,” and its involvement in gene expression has led to its recognition as an epigenetic agent.

Neurons, the principal cell type of the nervous system, are distinct in their ability to change their morphology, gene expression programs, and functions by adapting to their environment (Dotti et al., 1988; Arimura et al., 2004; Takano et al., 2015). This fundamental process is referred to as neuronal plasticity. It enables neurons to perform primary brain functions, such as learning and memory. Neurons acquire this property during maturation without cell division after having been committed to a cell type lineage. They intriguingly retain this ability for their exceptionally long lifespans. In the central nervous system (CNS), glial cells, such as astrocytes and oligodendrocytes, physically, metabolically, and functionally support neurons (Suzuki et al., 2011). Although neurons and glial cells both differentiate from common neural precursor cells (NPCs), their nuclear architecture has been shown to be distinct from one another (Takizawa and Meshorer, 2008). This observation implicates the importance of nuclear architecture in brain development and function.

We herein review a series of studies that demonstrate the functions and significance of nuclear architecture in the nervous system and discuss its relevance to brain function. The findings on post-mitotic neurons are especially intriguing, and therefore, understanding the underlying mechanisms of cell-division-independent rearrangement of nuclear architecture provides insight into the basic mechanisms of nuclear architecture.

## Nuclear Geometry

**FIGURE 1:**
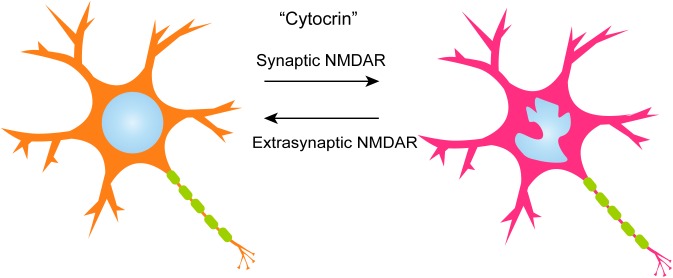
A model of activity-dependent nuclear infolding in rat hippocampal neurons. During baseline synaptic activity, neuronal nuclei display only minor infoldings if any at all. Initiation of strong electrical activity through the synaptic NMDA receptor leads to nuclear infolding, which then enhances nuclear calcium signaling and changes in gene expression. This change is reversed by the activation of the extrasynaptic NMDAR signaling pathways.

Along with the architecture inside the nucleus, the morphology of the organelle itself changes during neural differentiation and post-mitotic maturation. By analyzing three-dimensional reconstruction images of rat hippocampal neurons, Wittmann et al. (2009) found that a number of nuclei feature different degrees of infolding. Synaptic *N*-methyl-D-aspartate (NMDA) receptor activation dramatically increases the number of infolded nuclei. This observation is reversed when extrasynaptic NMDA receptors are activated by the bath-application of NMDA to the culture. Infolding increases the surface area of the nucleus, as well as the number of nuclear pore complexes. This further leads to an enhancement of nuclear calcium signaling especially in the smaller compartments of nuclei. Intriguingly, similar invaginations of the nuclear envelope (NE) have been observed in many types of mammalian cells (Fricker et al., 1997). An additional observation by Wittmann et al. (2009) supports the functional link between nuclear geometry and transcriptional regulation. Namely, synaptic-activity-induced phosphorylation of histone H3 on serine 10 is more robust in neurons with infolded nuclei. Invagination of the NE and subsequent rearrangement of the nuclear architecture may thus lead to changes in chromatin structure and activity-dependent gene expression. Although the precise mechanisms that regulate infolding are still unknown, the study suggests that changes in nuclear geometry and its regulation by extracellular inputs may play an important role in neuronal functions. These observations likely represent a “cytocrin system” in the nuclear architecture, of which an original concept has been proposed by Navarro et al. (2017) in the canonical epigenetics: extracellular factors could change nuclear geometry and cell functions (**Figure 1**).

## Nuclear Bodies

**FIGURE 2:**
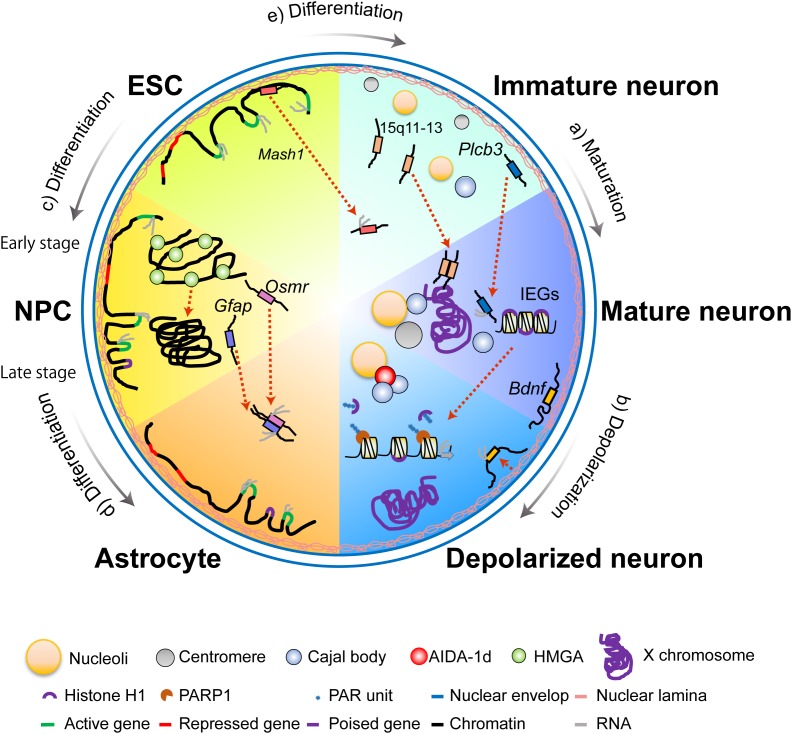
Divided views of nuclear architecture of different types of cells in the nervous system. **(a)** During the maturation process of neurons, repositioning of nucleoli from the nuclear periphery to the nuclear interior occurs alongside the relocation of Cajal bodies (CBs) and centromeric domains. Homologous pairing of the 15q11–13 imprinted domains and repositioning of the *Plcb3* gene loci are also observed in neuronal maturation. **(b)** Upon neuronal depolarization, the X chromosome migrates from the nuclear interior to the nuclear periphery. On the other hand, *Bdnf* gene loci move slightly away from the nuclear periphery. This shift is concurrent with transcriptional upregulation. Nuclear depolarization also features PARP1-dependent eviction of the linker histone H1 from immediate early genes and AIDA-1d-mediated association between CBs and nucleoli enable rapid transcription and protein synthesis. **(c,d)** During subsequent differentiation of the mouse ESC via a lineage-committed neural precursor cell (NPC) into terminally differentiated astrocytes, genome-nuclear lamina (NL) interactions change dramatically. Illustrated are five genes representative of different genome-NL movements. The housekeeping genes constantly move away from the NL and are maintained in the active state (green→green→green). The cell-cycle-related genes move away from NL in ESC and NPC but relocate to the NL in the astrocytes, which induces transcriptional downregulation (green→green→red). An ESC-specific gene relocates from the nuclear interior in the ESC to the NL in the NPC and the astrocyte, which lead to transcriptional downregulation (green→red→red). The NPC- specific genes relocate from the NL in the ESC to the nuclear interior in the NPC and astrocytes, but the genes maintain a poised state in the astrocyte (red→green→purple). The astrocyte-specific genes relocate from the NL in the ESC to the nuclear interior in the NPC and the astrocyte, but the genes maintain a poised state in the NPC (red→purple→green). Over the course of the differentiation, NPCs undergo an early-stage (neurogenic) to late-stage (astrogenic) transition, and downregulation of HMGA is followed by global chromatin condensation. Gene clustering between *Gfap* and *Osmr* occur with accompanying transcriptional upregulation of both genes during differentiation of the NPC into the astrocyte. **(e)** Upon neural induction from the embryonic stem cell (ESC), *Mash1* gene loci relocate from the nuclear periphery toward the nuclear interior, concomitant with transcriptional upregulation.

The arrangement of nuclear bodies is related to the functions of post-mitotic neurons. Nucleoli are found adjacent to the nuclear periphery in immature Purkinje neurons but converge into one or two larger nucleoli while relocating toward the center of the nucleus during maturation (**Figure 2a**; Solovei et al., 2004). This rearrangement is likely associated with normal brain development and function, as it depends on methyl CpG binding protein 2 (MeCP2). Mutations of this protein cause a neurological disorder known as Rett syndrome (Singleton et al., 2011).

Cajal bodies, which are speculated to act as sites of assembly for small nuclear ribonucleoproteins, are often found adjacent to nucleoli in mature neurons (**Figure 2a**; Pena et al., 2001; Casafont et al., 2006). The number of CBs increases during neuronal differentiation (Janevski et al., 1997) and is closely related to transcriptional activity after differentiation (Santama et al., 1996; Pena et al., 2001). Investigations using primary rat hippocampal neurons have revealed that CBs mediate a direct link between synaptic activity and nuclear bodies (Jordan et al., 2007). Upon stimulation of NMDA receptors, a component of postsynaptic density, AIDA-1d translocates into the nucleus and binds to CBs (**Figure 2b**). This binding further modulates the number of nucleoli and global protein synthesis.

Although PML bodies have been implicated in a variety of cellular processes, ranging from transcription and cell-cycle progression to apoptosis and DNA repair, their precise role has yet to be defined. Investigations have, however, elucidated a putative role of PML bodies in regeneration and axonotomy; although the number of PML bodies decreases during neural differentiation (Aoto et al., 2006), the quantity is reportedly increased in dorsal root ganglion neurons of patients with acute inflammatory demyelinating polyneuropathy (Villagrá et al., 2004).

Taken together, these data demonstrate a close relationship between the arrangement of nuclear bodies and neural differentiation/maturation, as well as the importance of the former to neuronal function. The underlying mechanisms that regulate the arrangement of nuclear bodies have not been fully elucidated as of yet. The studies from this point of view will provide a new venue for comprehension of neural differentiation, maturation, and neuronal activity.

## Chromosomes and Their Specific Regions

In the interphase cell nucleus, chromosomes adopt a conserved, non-random arrangement in subnuclear domains called chromosome territories (CTs), which change according to cell differentiation and functions (Cremer and Cremer, 2010). Several studies on neurons during and after maturation demonstrate that positions of CTs and specific genome regions of chromosomes are not static in the interphase nucleus, as they can change via cell-division-independent mechanisms. A prominent example was identified by Barr and Bertram’s (1949) seminal study. Upon electrical stimulation, the nucleolar satellite (i.e., the Barr body or the inactive X chromosome) in cat motor neurons moves from its usual position adjacent to the nucleolus toward the nuclear membrane (**Figure 2b**; Barr and Bertram, 1949). By examining epileptic human cortices, Borden and Manuelidis (1988) built upon the former observation by finding a dramatic repositioning of the centromeric heterochromatin region of the X chromosome in neurons within electrophysiologically defined seizure foci in both men and women. These studies suggest that specific CTs reposition in a manner that is dependent on neuronal activity and independent of cell division.

The repositioning of specific regions of a chromosome in post-mitotic neurons has also been reported. In mouse Purkinje neurons, the number of centromeric domains changes during maturation, along with their shift from the nuclear periphery to the nucleolus (**Figure 2a**; Martou and De Boni, 2000). This spatial redistribution may contribute to differentiation by placing specific genome sequences onto transcriptionally competent nuclear sites. In addition, the number of centromeric domains is reduced in tetanized rat hippocampal CA1 neurons relative to unpotentiated neurons (Billia et al., 1992). Given that a similar decrease was also observed in CA1 neurons exposed to NMDA, these data suggest that the repositioning of centromeric domains is involved in neuronal-activity-dependent large-scale transcription and protein synthesis.

Chromosome reorganization in post-mitotic neurons was further reported in a study on rod photoreceptor cells of different species. The vast majority of eukaryotic nuclei display a consistent nuclear architecture. Euchromatin predominantly occupies internal nuclear regions and heterochromatin is primarily found on the surface of the NE in regions devoid of nuclear pores and on the surface of the nucleolus. In nocturnal mammals, however, the positions of euchromatin and heterochromatin in the nuclei of rod photoreceptor cells are inverted to reduce light loss and collect it efficiently (Solovei et al., 2009). The low expression of both lamin A/C and lamin B receptor (LBR) underlies this inversion. Cells with conventional nuclear architecture express high levels of these proteins (Solovei et al., 2013). Similar to what is observed in rod photoreceptor cells, low expression of LBR in olfactory sensory neurons (OSNs) contributes to the aggregation of inactive olfactory receptor (OR) genes (Clowney et al., 2012). Further, the expression level of LBR has been observed to decrease during neuronal differentiation from NPCs (Bonev et al., 2017). The aforementioned findings may implicate the composition of the inner nuclear membrane (INM) in chromosome reorganization of post-mitotic neurons and the cell fate of NPCs, and the verification of this possibility may uncover new mechanisms of NPC differentiation.

## Chromatin

Global structures of chromatin also change during neural differentiation and maturation. One of the structural features of global chromatin organization is the spacing between two adjacent nucleosomes, termed the nucleosome repeat length (NRL), which has been shown to change in rat cortical neurons during neuronal differentiation and maturation (Ermini and Kuenzle, 1978; Jaeger and Kuenzle, 1982). In addition to NRL, global chromatin packaging states are important for the differentiation competence of NPCs (Kishi et al., 2012). Over the course of differentiation, NPCs undergo an early-stage (neurogenic) to late-stage (astrogenic) transition. HMGA proteins, which are highly expressed in NPCs only during the former phase, are essential to the global opening state of chromatin and the neurogenic competency of NPCs (**Figures 2c,d**).

Global changes in chromatin organization are also important for transcriptional upregulation of activity-dependent immediate early genes (IEGs) in post-mitotic neurons. Chromatin accessibility is an indicator of global chromatin change. An investigation studied this property in adult mouse dentate granule neurons by subjecting them to synchronous neuronal activation using transposase-accessible chromatin *in vivo* via a sequencing assay (ATAC-seq). Changes elicited by the procedure were observed by comparing the neurons before and after the activation (Su et al., 2017). This study found that neuronal activity leads to genome-wide chromatin opening and observed that gained-open sites are enriched at active enhancer regions and binding sites for AP1-complex components, including *c-Fos*. Although this study suggests that *c-Fos* is implicated in initiating, but not maintaining, neuronal-activity-dependent chromatin opening, precise mechanisms underlying *c-Fos* activity remain elusive. A recent study, however, demonstrated that potassium chloride-induced depolarization of mouse cortical neurons causes a rapid release of linker histone H1 from chromatin and an increase in IEG expression, including *c-Fos* (**Figure 2b**; Azad et al., 2018). Poly-ADP ribosylation and phosphorylation of H1 are essential to these two processes. An investigation has shown that administration of a PARP-1 inhibitor impedes long-term memory formation in Aplysia (Cohen-Armon et al., 2004). This observation is further supported by the finding that DNA from Alzheimer’s disease (AD) patients is less sensitive to micrococcal nuclease - an enzyme that digests chromatin in the linker region between two adjacent nucleosomes - than that from patients with other neuronal disorders (Lewis et al., 1981; Lukiw and Crapper McLachlan, 1990). These studies therefore suggest the importance of *c-Fos* in the global control of chromatin organization, as well as the therapeutic potential of PARP-1 activity modulation in treating neurocognitive diseases via changing chromatin states.

In addition to linker histone H1, an additional global chromatin organizer CCCTC-binding factor (CTCF) features important roles in neuronal development (see review Davis and Elliott, 2018). In the adult mouse brain, a conditional knockout (CKO) of CTCF in excitatory forebrain neurons yields specific deficits in learning and memory, including spatial memory and fear memory (Sams et al., 2016). Genes involved in memory and learning functions, such as *Arc* and *brain-derived neurotrophic factor* (*Bdnf*), are downregulated in CTCF CKO hippocampi. A chromosome confirmation-capture-based (3C-based) analysis showed that this transcriptional abnormality is attributed to the loss of CTCF-dependent chromatin interaction within these genes loci. Further research has supported such findings: CTCF is mutated in a subset of individuals with intellectual disabilities (Gregor et al., 2013) and its binding partners such as cohesion (Wendt et al., 2008), CHD8 (Ishihara et al., 2006; O’Roak et al., 2012), and MeCP2 (Kernohan et al., 2014) have been implicated in neurodevelopmental disorders. These results suggest that targeting the abnormal functioning of CTCF and its binding partners may be an effective therapeutic strategy for treating neurodevelopmental disorders.

Among these CTCF-binding partners, MeCP2 has been shown to be involved in neuronal chromatin organization (see review Ausio et al., 2014). At first, MeCP2 was only recognized as a transcriptional repressor because it contains a methyl-CpG binding domain and a transcriptional repression domain. However, it has been found that MeCP2 is distributed widely across the genome of neurons (Skene et al., 2010), and its depletion leads to both an increase and a decrease in gene expression in the brain (Chahrour et al., 2008; Skene et al., 2010). In addition, MeCP2 binds to linker DNA and can displace linker histone H1 (Ghosh et al., 2010). These studies suggest that MeCP2 affects the structure of chromatin much like the linker histone H1. Despite their similarity, a recent study indicated that they work independently in chromatin binding (Ito-Ishida et al., 2018). Another global chromatin organizer, HMGA proteins, also competes with H1 to bind with chromatin (Kishi et al., 2012). Further studies on the functional relationship among these global chromatin organizer proteins in neurons will help to uncover the molecular basis of neurodevelopmental disorders.

## Specific Gene Loci

Along with global changes, local repositioning of specific gene loci correlates with the transcriptional activity in neural cells. Studies on this topic have operated from two distinct points of view, radial gene positioning and gene clustering.

### Radial Gene Positioning

Akin to chromosomes and their specific regions, gene loci are non-randomly arranged within the mammalian nucleus. The radial repositioning of gene loci away from the nuclear periphery correlates with the transcriptional upregulation of genes during neural differentiation and development. The nuclear lamina, a protein meshwork located along the inner layer of the nuclear membrane, interacts with repressive chromatin regulators to tether heterochromatic sequences to the nuclear periphery. Indeed, an important proneural regulator gene, *Mash1*, is repositioned from the nuclear periphery toward the nuclear interior (**Figure 2e**). This migration is concurrent with the changes in chromatin structure and transcriptional upregulation upon neural induction from ES cells (Williams et al., 2006). Further support for the association between radial gene positioning and transcriptional upregulation has been observed in the maturation of mouse cerebellar Purkinje neurons. The repositioning of *Plcb3* from the nuclear periphery toward the nuclear interior is concomitant with transcriptional upregulation (**Figure 2a**; Martou et al., 2002). Although the relationship between the two processes is still under investigation, an increasing body of evidence suggests the interaction between the genome and the nuclear lamina is important for normal brain development and function. Intriguingly, lamina-associated heterochromatic domains became mobile during brain development. Peric-Hupkes et al. (2010) revealed this mechanism by performing DNA adenine methyltransferase identification (DamID) of Lamin B1 to map lamina-associated domains (LADs) during the subsequent differentiation of mouse embryonic stem cells via lineage-committed NPCs into terminally differentiated astrocytes (**Figures 2c,d**). Though LADs overlapped by 73–87% among the examined cell types, many gene loci that determine cellular identity relocate from the lamina. Several such genes that move away from the lamina are concurrently activated, while others become activated in the next phase of differentiation. Furthermore, most gene loci apparently relocate separately, not as clusters, and many gene loci migrate according to cell type, differentiation stage, and the expression levels of the genes. This mechanism may compose another layer of spatial-gene-positioning-related transcriptional regulation during neuronal differentiation. Studies using rat hippocampal dentate gyrus neurons have further found that intranuclear positions of *Bdnf* move away from the nuclear lamina upon neuronal activation following kainite-induced seizures (**Figure 2b**; Walczak et al., 2013). These results demonstrate that the genome-nuclear lamina interaction plays an important role in brain function.

Radial positioning of genes is also implicated in cell fate of NPCs. In *Drosophila* embryonic NPCs, Hunchback (Hb) is expressed in the early stage NPCs and is both necessary and sufficient to specify early-born neuronal identity. Interestingly, gene loci of *Hb* move toward the nuclear periphery when competence to specify early-born fates is lost (Kohwi et al., 2013). As Hb expression can be induced when artificially placed in the nuclear interior, this repositioning seems to be required to efficiently and permanently silence Hb transcription. In mouse NPCs, an astrocyte-specific gene encoding *glial fibrillary acidic protein* (*Gfap*) is repositioned toward the nuclear center during astrocyte differentiation (Takizawa et al., 2008). Following its relocation to the nuclear interior, the active *Gfap* allele associates more frequently with nuclear speckles, a nuclear body enriched in pre-mRNA splicing factors. These results represent dynamic changes in the radial distribution of specific gene loci followed by concomitant alterations in transcriptional activity upon differentiation from NPCs.

### Gene Clustering Between Gene Loci

An increasing amount of evidence supports the importance of gene clustering to biological processes, including transcriptional regulation and enhancement (Schoenfelder et al., 2010). By using a chromosome confirmation-capture-based (3C-based) technique and an expression array, 18 genes were identified to specifically associate with an astrocyte-specific gene, *Gfap*, in NPC-derived astrocytes (Ito et al., 2016). *Gfap* expression is induced by activation of transcription factor STAT3 (Takizawa et al., 2001). Given that several of the identified genes are also activated by the same transcription factor, their transcription may be co-regulated with *Gfap* on the same locale of the nucleus. *Osmr*, one of the 18 putative clustering genes, encodes the oncostatin M receptor (OSMR). Clustering of *Osmr* with *Gfap* enhances the transcription of both genes and requires the presence of both brahma-related gene 1 (BRG1), an ATP-dependent chromatin remodeling factor, and STAT3 (**Figure 2d**; Ito et al., 2018). Further, an investigation of mouse neural differentiation using ultrahigh resolution Hi-C, a 3C-based technique, revealed the involvement of cortical-neuron (CN)-specific transcription factors, such as Pax6, NeuroD2, and Tbr1, in CN-specific long-range chromatin interaction both *in vivo* and *in vitro* (Bonev et al., 2017). These studies demonstrate that transcription factors, which have been shown to be important for cell fate determination of NPCs, are involved in brain development via gene clustering.

Gene clustering between homologous gene loci has further been reported to contribute to gene regulation (Hogan et al., 2015). Homologous pairing of the 15q11–13 imprinted domains, which are deficient in maternal alleles in Angelman syndrome (AS), is observed in infantile and juvenile normal human brains (**Figure 2a**). However, it is not observed in brain samples derived from patients with AS, Rett syndrome, autism, or other conditions (Thatcher et al., 2005). The finding implicates gene clustering between homologous gene loci in pathophysiological states. The function and importance of long-range chromatin interactions, such as promoter-enhancer interaction within gene bodies or topologically associating domains in neural gene expression, is becoming increasingly well understood (see an excellent review Rajarajan et al., 2016). However, the mechanism and significance of gene clustering between gene loci in brain development and function are still unknown and require extensive investigation.

## Conclusion and Future Direction

Epigenetic changes are accompanied by the reconstruction of nuclear architecture. The proteins that participate in the construction of nuclear architecture, such as cohesin, NIPBL, MeCP2, CTCF, SATB2, and LMNB1, have been shown to be pivotal for a healthy nervous system. The dysfunction of these molecules reportedly causes neurodevelopmental disorders (see excellent review Rajarajan et al., 2016).

Neurons consist of many subtypes of cells, and this diversity underlies the intricate functions of the nervous system. Nuclear architecture is presumably distinct among such subtypes. To elucidate the differences in their nuclear architecture at a single-cell level and to characterize their components, such as genome-nuclear lamina interaction and chromatin conformation, novel methods have been developed (Kind et al., 2013; Nagano et al., 2013). In addition, *in vivo* studies are increasingly performed to characterize the natural states of nuclear architecture and avoid the limitations of *in vitro* approaches (Bonev et al., 2017). At the forefront of such *in vivo* investigations, the CRISPR/Cas9 system and its derivative technologies have been applied to studies of epigenetic states, such as DNA methylation and chromatin conformation (Guo et al., 2015; Liu et al., 2016; Morita et al., 2016). Although conducting single-cell-based analyses of nuclear architecture and gene expression *in vivo* poses difficulties, overcoming these experimental challenges will open a new avenue for uncovering the importance of nuclear architecture and its significance to brain function in diseased and healthy states.

## Author Contributions

All authors listed have made a substantial, direct and intellectual contribution to the work, and approved it for publication.

## Conflict of Interest Statement

The authors declare that the research was conducted in the absence of any commercial or financial relationships that could be construed as a potential conflict of interest.
